# Differential germination characteristics of glyphosate-resistant and glyphosate-susceptible *Chloris virgata* populations under different temperature and moisture stress regimes

**DOI:** 10.1371/journal.pone.0253346

**Published:** 2021-06-17

**Authors:** Het Samir Desai, Bhagirath Singh Chauhan

**Affiliations:** 1 Queensland Alliance for Agriculture and Food Innovation (QAAFI), The University of Queensland, Gatton, Australia; 2 School of Agriculture and Food Sciences (SAFS), The University of Queensland, Gatton, Australia; Brigham Young University, UNITED STATES

## Abstract

Thorough knowledge of the germination behavior of weed species could aid in the development of effective weed control practices, especially when glyphosate resistance is involved. A study was conducted using two glyphosate-resistant (GR) (SGW2 and CP2) and two glyphosate-susceptible (GS) (Ch and SGM2) populations of *Chloris virgata*, an emerging and troublesome weed species of Australian farming systems, to evaluate their germination response to different alternating temperature (15/5, 25/15 and 35/25°C with 12 h/12 h light/dark photoperiod) and moisture stress regimes (0, -0.1, -0.2, -0.4, -0.8 and -1.6 MPa). These temperature regimes represent temperatures occurring throughout the year in the eastern grain region of Australia. Seeds germinated in all the temperature regimes with no clear indication of optimum thermal conditions for the GR and GS populations. All populations exhibited considerable germination at the lowest alternating temperature regime 15/5°C (61%, 87%, 49%, and 47% for Ch, SGM2, SGW2, and CP2, respectively), demonstrating the ability of *C*. *virgata* to germinate in winter months despite being a summer annual. Seed germination of all populations was inhibited at -0.8 and -1.6 MPa osmotic potential at two alternating temperature regimes (15/5 and 35/25°C); however, some seeds germinated at 25/15°C at -0.8 MPa osmotic potential, indicating the ability of *C*. *virgata* to germinate in arid regions and drought conditions. Three biological parameters (T10: incubation period required to reach 10% germination; T50: incubation period required to reach 50% germination; and T90: incubation period required to reach 90% germination) suggested late water imbibition with increasing moisture stress levels. The GR population SGW2 exhibited a distinctive pattern in T10, T50, and T90, possessing delayed germination behaviour and thus demonstrating an escape mechanism against pre-plating weed management practices. Knowledge gained from this study will help in developing site-specific and multi-tactic weed control protocols.

## Introduction

*Chloris virgata* Sw. is a summer annual species of Poaceae family that is considered a troublesome weed species across the tropics of the world. It possesses a C_4_ photosynthesis mechanism, monocotyledon seed structure, and is commonly referred to by many names depending on the region, such as Feathertop Rhodes grass in Australia; feathered finger grass, feathered windmill grass, and feathered Chloris in the United States; and oldland grass and sweetgrass in south-east Asia [1]. It can reach more than 1 m height, produce 600 g m^-2^ dry matter under suitable (summer) weather conditions [2], and has been classified as a host for aphids, barley yellow dwarf and cereal yellow dwarf virus, and some species of nematodes [3].

*Chloris virgata* can produce more than 40,000 seeds plant^-1^ and is therefore considered a prolific seed producer [2]. The seeds of *C*. *virgata* are comparatively smaller than other weed species, aerodynamic in shape, lightweight, and exhibit two protruded trichomes (hairs). These morphological structures aid seeds in dispersal from one ecosystem to another and increase *C*. *virgata*’s potential invasiveness [4]. An earlier study suggested that the seeds of *C*. *virgata* can travel up to 13 m in normal wind velocity [1]. Moreover, the seeds of *C*. *virgata* prefer two kinds of dispersal methods: hydrochory and anemochory (dispersal through water and wind, respectively). The two protruded trichomes could also help *C*. *virgata* seeds in sticking to agricultural machinery and labors, thus reducing the energy required for dispersal.

*Chloris virgata* is known to have originated from the warm regions of the world; however, the exact origin of this species is yet to be identified and is open for international debate [5]. It is recognized as an emerging and problematic cropping weed species in Australian farming systems. This species is greatly distributed across the mainland of Australia and is considered as a major weed species of South Australian vineyards, grain cropping regions of Queensland, Western Australia, and Northern Territory, and cotton-growing regions of New South Wales [1]. The widespread occurrence of this species has been observed in an area of 118,000 ha of Australian agricultural land and is responsible for a substantial amount of yield and revenue loss equalling 39,300 tons of grain and AUD 7.7 million per annum, respectively [6]. A study conducted at the University of Queensland suggested that 22–25 plants m^-2^ of *C*. *virgata* reduced the mungbean yield by 50% compared to a weed-free treatment plot [7]. Considering the economic impact of this species on Australian agriculture, it has been listed among the top 20 weeds of major concerns in Australia [6].

The shift from traditional agriculture to conservation agriculture offers substantial benefits to Australian farming systems, such as less energy-exhaustive farming practices, less environmental degradation, and a higher margin of profits compared to traditional farming systems [8]. However, several species, which were previously considered as environmental weeds, have established in Australian agroecology due to the adoption of conservation agricultural practices (e.g., no-till systems) as well as their peculiar biology, rapid dispersal ability, reproductive potential, and efficient competitiveness [9,10]. Of these weed species, *C*. *virgata* is one of the most important examples. The long-term use of no-till systems drastically changed weed management practices and caused over-reliance on glyphosate-based weed control. Owing to the long-term usage of glyphosate-based weed management operations in summer fallows and glyphosate-tolerant cotton, several weed species have developed resistance against glyphosate, including *C*. *virgata*. After the first case of glyphosate resistance of *C*.*virgata* in Australia in 2015, several populations of this species have been identified as glyphosate-resistant (GR) [4].

Seed germination ability is an essential phenomenon in identifying the efficiency of any weed species to establish successfully in agroecosystems [11]. Germination processes are known to be regulated by several environmental factors, such as temperature, soil moisture, soil pH level, light intensity, and photoperiod [12]. However, temperature, soil moisture, and photoperiod are considered the most impactful environmental factors on germination [13]. In previous studies on several weed species, it was observed that GR and glyphosate-susceptible (GS) populations possessed different germination characteristics [14–18].

The present study was conducted to understand the differential germination response of two GR and two GS populations of *C*. *virgata* to different temperature regimes and moisture stress regimes. In order to reduce the over-dependence on chemical weed control tactics and develop more effective weed control programs by adopting integrated weed management operations, detailed knowledge of seed germination biology would offer opportunities to achieve management of competitive weed species, such as *C*. *virgata*. The major aim of this study was to enhance the scant knowledge about *C*. *virgata* germination in response to different temperature regimes and moisture stress regimes, whether this differs between GR and GS populations, and different water stress adaptability of GR and GS populations under different alternating temperature regimes. The data from this study will aid weed scientists and agronomists to develop an effective weed control program for this emerging weed species.

## Materials and methods

### Seed collection

Seeds of *C*. *virgata* were collected from multiple sites across the grain-growing regions of Queensland, Australia in March-April 2017. Initially, 10 populations were collected from five locations [4] and later screened with different doses of glyphosate to identify GR and GS populations. All populations were given precise and unique coding to keep records for future collection. Mature panicles were detached by using garden secateurs and immediately placed in individual paper bags according to population. Also, seeds were collected from several plants within the population to acquire seed lot representatives. Paper bags were also labeled according to the coding given to all populations. The whole collected seed lot was then transported to the Queensland Alliance for Agriculture and Food Innovation (QAAFI) weed science laboratory at the University of Queensland, Gatton (latitude -27.5551 and longitude 152.3343) where all the seeds were separated from panicles manually and stored in airproof containers to avoid unwanted contamination. All the containers were stored at room temperature (20–25°C) in dark conditions [19]. Seed production was carried out as described by [4].

### Seed viability test

It was earlier hypothesized that black-colored seeds are more viable than the ivory-colored seeds for this particular species [20]. However, according to our previous observations, not all black-colored seeds are viable. Consequently, all the seeds were subjected to the X-raying technique.

Black and white seeds of individual populations were placed in the Petri dish over the filter paper. A desk-mounted magnifying glass was used for seed placement. Prepared Petri dishes were then placed on the platform of the X-ray machine (Faxitron MX-20 X-ray machine, Australia) in order to get X-ray images of seeds. Non-viable and hollowed seeds were removed by forceps. In this way, four different seed batches (one for each population) were prepared with viable seeds for the germination experiment.

### Experimental approach and design

A laboratory experiment was conducted to assess the effect of various alternating temperature regimes and moisture stress levels using a split-plot design in which alternating temperature regimes were the main plot factors and moisture stress regimes were the subplot factors. Three alternating temperature regimes (15/5°C, 25/15°C, and 35/25°C with 12 h/12 h light/dark photoperiod) and six moisture stress regimes (0, -0.1, -0.2, -0.4, -0.8, and -1.6 MPa) were employed to evaluate the germination response of GR (SGW2 and CP2) and GS (Ch and SGM2) populations of *C*. *virgata*.

Moisture stress treatments were replicated three times within each alternating temperature treatment. Three different temperature regimes were provided by three different incubators (Labec Laboratory Pvt. LTD., Australia) in which a temperature regulator connected to a data logger (Invensys Eurotherm, 3216 PID temperature controller) was installed to monitor alternative temperature regimes. A photoperiod of 12 h/12 h light/dark was controlled by fluorescent lamps (Ultralamp ECO-T5, 28W, 1170 mm) installed in all three incubators. Moreover, the flush mount time clock (Grasslin Uni 45 series timers) installed in all three incubators was used to regulate the timing of alternating temperature regimes and photoperiod. Moisture stress treatments of 0, -0.1, -0.2, -0.4, -0.8, and –1.6 MPa were developed by dissolving an appropriate quantity of polyethylene glycol (PEG) into deionized water, corresponding to different alternating temperature regimes according to Eq 1 [21]:

$$\Psi \text{s}=0.013{\left(\text{PEG}\right)}^{2}\text{T}–13.7{\left(\text{PEG}\right)}^{2}$$ (1)

where ‘PEG’ denotes the quantity of polyethylene glycol (g PEG g^-1^ water) and ‘T’ denotes the temperature.

### Germination measurements

A germination experiment was carried out on two GR (SGW2 and CP2) and GS (Ch and SGM2) populations at the QAAFI weed science laboratory, Gatton, Queensland, Australia in 2020. Double filter papers (Macherey-Nagel GmbH & Co. Kg, Germany) were placed in each Petri dish (92 mm diameter × 16 mm height). All the filter papers installed in Petri dishes were moistened by adding 5 ml of deionized water (control treatment) or 5 ml PEG solution corresponding to the treatments using a micropipette. Twenty-five seeds of each population were placed uniformly in each Petri dish using a pair of forceps and a desk mount LED laboratory magnifier lamp (White label, Model: QM3546) was used for seed placement considering the small seed size of *C*. *virgata*. Zip-lock plastic bags were used to hold Petri dishes.

Seed germination counts were observed daily, and germinated seeds were removed using a pair of forceps. Final germination counts were then converted into the final cumulative germination (FCG). Seeds were considered to have germinated once radicle emergence was visible (>1 mm). Evaporation was high for Petri dishes under 35/25°C temperature regime; therefore, to maintain constant moisture stress conditions, 2 ml of deionized water or PEG solutions were added every 2 d in all the Petri dishes at this temperature regime. The germination experiment was considered complete after no germination was observed for five consecutive days.

### Statistical analysis

The experiment was carried out in a split-plot design with three replications. The FCG data set was subjected to two-way analysis of variance (ANOVA) with multiple comparisons in which means were separated by Fisher’s LSD test (GraphPad 8.4.2 679. GraphPad Software Inc., California) to observe the significant difference of individual treatment. Data transformation did not improve the homogeneity of variance; therefore, non-transformed data were used for ANOVA. Percentage germination reduction of the control treatment (0 MPa) within each alternating temperature range of each population was calculated manually and further fitted using a three-parameter sigmoidal model (Eq 2) (Sigmaplot 14.0, Systat Software Inc., San Jose, California, USA) to understand the stress adaptability of the populations at different alternating temperature regimes.


$$f=a/\left(1+exp{\left(\frac{x}{x0}\right)}^{b}\right)$$ (2)

Codifications: *‘f’*: final cumulative germination; *‘a’*: maximum germination percentage (maximum number on Y-axis or inflection point); ‘*x0*’: osmotic potential level corresponding to a 50% germination reduction; and *‘b’*: the slope of regression curve.

The ‘SeedCalc’ package in R software was used to determine the incubation period required to attain 10%, 50%, and 90% germination (T10, T50, and T90, respectively) [22].


$$T10=\frac{ti+\left\{\left[\frac{N}{\frac{100}{10}}\right]-ni\right\}\left(tj-ti\right)}{\left(nj-ni\right)}$$ (3)

Codification: *‘N’*: total number of germinated seeds; *‘ni’* and *‘nj’*: final number of germinated seeds in adjacent counts of time *‘ti’* and *‘tj’*, respectively.


$$T50=\frac{ti+\left\{\left[\frac{N}{\frac{100}{50}}\right]-ni\right\}\left(tj-ti\right)}{\left(nj-ni\right)}$$ (4)

Codifications: As mentioned for *‘T10’*.


$$T90=\frac{ti+\left\{\left[\frac{N}{\frac{100}{90}}\right]-ni\right\}\left(tj-ti\right)}{\left(nj-ni\right)}$$ (5)

Codifications: As mentioned for *‘T10*.

## Results and discussion

Osmotic potential (MPa) had a significant influence on the germination of all four populations at the three alternating temperature regimes (p<0.001 for all temperatures) (Table 1). The interaction of temperature and population was non-significant. The FCG of the GR populations (SGW2 and CP2) did not exceed more than 60% at any temperature regime. At 0 MPa osmotic potential level, germination was 49% and 47% for SGW2 and CP2, respectively, at 15/5°C; 60% and 37% for SGW2 and CP2, respectively, at 25/15°C; and 39% and 43% for SGW2 and CP2, respectively, at 35/25°C (Table 1). On the other hand, FCG at 0 MPa was 61% and 87% for Ch and SGM2, respectively, at 15/5°C; 65% and 79% for Ch and SGM2, respectively, at 25/15°C; and 48% and 74% for Ch and SGM2, respectively, at 35/25°C (Table 1). These results suggest that the FCG of the GR populations (SGW2 and CP2) was lower compared to the GS populations (Ch and SGM2) (p = 0.001). This could be due to different dormancy levels of the GR and GS populations as only viable seeds were used for all populations. Similar results were found in *Bassia scoparia* (L.) A. J. Scott, in which the GR populations exhibited lower germination (ranging from 53% to 70%) than the GS populations (ranging from 95% to 100%) when tested at different alternating temperature regimes [18].

**Table 1:** Final cumulative germination of two glyphosate-susceptible (Ch and SGM2) and two glyphosate-resistant (SGW2 and CP2) populations of *Chloris virgata* in response to different temperature regimes and moisture stress levels.

Temperature (° C)	Osmotic Potential (MPa)	Glyphosate susceptible (GS)		Glyphosate resistant (GR)	
		Ch	SGM2	SGW2	CP2
15/5	0	61.3 ± 1.4 (0)	86.7 ± 9.6 (0)	49.3 ± 2.5 (0)	46.7 ± 5.3 (0)
	-0.1	42.7 ± 3.5 (30)	69.3 ± 7.0 (20)	41.3 ± 8.1 (16)	26.7 ± 7.0 (43)
	-0.2	40.0 ± 10.1 (35)*	50.7 ± 1.3 (42)	34.7 ± 10.4 (30)	22.7 ± 3.5 (51)*
	-0.4	8.0 ± 2.0 (87)*	24.0 ± 6.1 (72)*	12.0 ± 4 (76)	16.0 ± 6.1 (66)*
	-0.8	0 ± 0 (100)*	0 ± 0 (100)*	0 ± 0 (100)*	0 ± 0 (100)*
	-1.6	0 ± 0 (100)*	0 ± 0 (100)*	0 ± 0 (100)*	0 ± 0 (100)*
25/15	0	65.3 ± 1.7 (0)	78.7 ± 2.7 (0)	60.0 ± 2.3 (0)	37.3 ± 8.7 (0)
	-0.1	54.7 ± 3.5 (16)	69.3 ± 8.1 (12)	44.0 ± 4.0 (27)	34.7 ± 1.3 (7)
	-0.2	53.3 ± 7.0 (18)	64.0 ± 2.3 (19)*	41.3 ± 4.8 (31)*	29.3 ± 2.7 (21)
	-0.4	50.7 ± 3.5 (22)	48.0 ± 2.3 (39)*	28.0 ± 6.1 (53)*	25.3 ± 5.3 (32)
	-0.8	1.3 ± 1.3 (98)*	2.7 ± 2.7 (97)*	0 ± 0 (100)*	0 ± 0 (100)*
	-1.6	0 ± 0 (100)*	0 ± 0 (100)*	0 ± 0 (100)*	0 ± 0 (100)*
35/25	0	48.0 ± 6.1 (0)	73.3 ± 3.5 (0)	38.7 ± 2.7 (0)	42.7 ± 3.5 (0)
	-0.1	44.0 ± 4.0 (8)	57.3 ± 4.8 (22)	37.3 ± 3.5 (4)	34.7 ± 2.7 (19)
	-0.2	40.0 ± 0 (17)	46.7 ± 7.0 (36)	26.7 ± 5.8 (31)	29.3 ± 7.0 (31)
	-0.4	24.0 ± 4.0 (50)*	25.3 ± 1.3 (66)*	22.7 ± 3.5 (41)	22.7 ± 2.7 (47)*
	-0.8	0 ± 0 (100)*	0 ± 0 (100)*	0 ± 0 (100)*	0 ± 0 (100)*
	-1.6	0 ± 0 (100)*	0 ± 0 (100)*	0 ± 0 (100)*	0 ± 0 (100)*

Values after ± and in parentheses are standard errors of means and % reduction of germination to control treatment, respectively. Asterisks denote a significant difference of individual treatment compared to control according to Fisher‘s LSD test at a 0.05% probability level.

The FCG of Ch and SGW2 was significantly higher (65% and 60%, respectively) under 25/15°C at 0 MPa osmotic potential compared to the other two alternating temperature regimes. Population SGM2 exhibited 79% FCG at 25/15°C which was lower than the FCG (87%) at 15/5°C; however, the difference was non-significant (p = 0.478). Similarly, the CP2 population possessed higher FCG at 15/5°C (47%) and 35/25°C (43%) compared to 25/15°C (37%), though the differences were non-significant. As mentioned earlier, *C*. *virgata* is a summer annual weed species. However, it tends to acclimatize rapidly in terms of germination [20]. FCG was higher for two populations (Ch and SGW2) under 25/15°C at 0 MPa compared to the other two temperature ranges. There was a non-significant difference in germination for the two populations (SGM2 and CP2) under all tested temperature regimes at 0 MPa. Moreover, germination of SGM2 was also considerable at 15/5°C (87%) and 35/25°C (73%). The overall germination of CP2 was lower compared to the other populations, still it exhibited about 40% germination at all tested temperature regimes. Therefore, these results suggest 25/15°C as an ideal temperature for optimum germination of *C*. *virgata* and the other two alternating temperature regimes (15/5°C and 35/25°C) can be considered sub-optimum thermal conditions for *C*. *virgata* in terms of germination.

**Fig 1:**
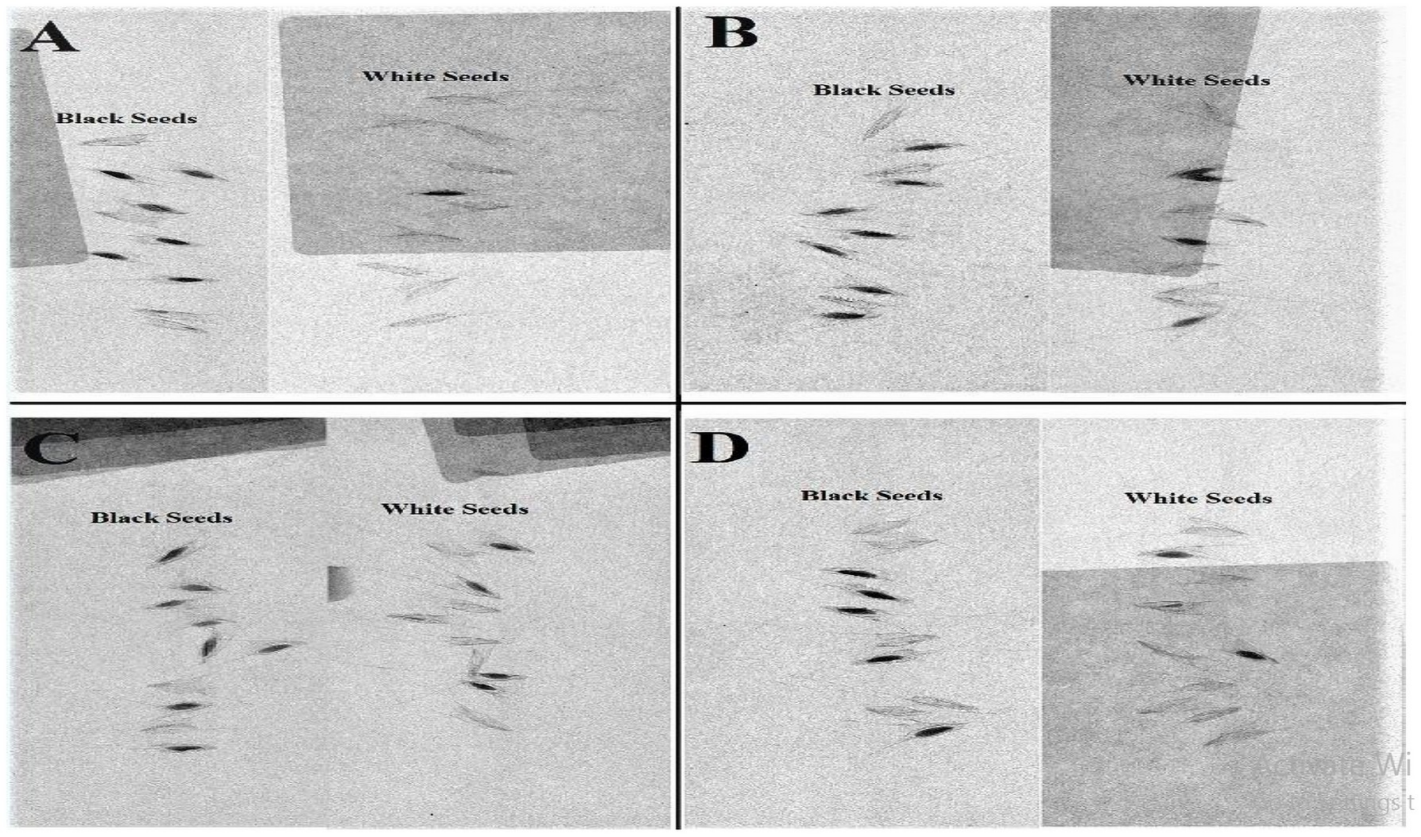
Reductions in germination percentage of two glyphosate-susceptible (Ch and SGM2) and glyphosate-resistant (SGW2 and CP2) populations of *Chloris virgata* at decreasing osmotic potential levels at different alternating temperature regimes. The estimated parameters were presented in Table 2.

FCGs of all populations decreased with decreasing osmotic potential (MPa) levels (Table 1); however, no considerable difference was observed in reduction percentages with decreasing osmotic potential levels between populations at all three alternating temperature regimes (Fig 1). All populations exhibited 0% germination at -0.8 and -1.6 MPa at two sub-optimal thermal conditions (15/5°C and 35/25°C). However, some seeds of the two GS populations (Ch and SGM2) germinated at -0.8 MPa in the optimal thermal conditions (25/15°C), indicating the germination ability of *C*. *virgata* even in arid regions and under drought conditions. Due to the susceptibility of Ch and SGM2 against glyphosate [4], these two populations can easily be controlled using the judicious use of glyphosate. However, the efficacy of glyphosate might be decreased when applied to weed species suffering from abiotic stress (e.g., water stress) [23]. The reduced efficacy could be because of less herbicide absorption and translocation as herbicides are largely translocated via vascular systems [24].

Reductions in germination percentages, compared to the control (0 MPa osmotic potential), were analyzed at all osmotic potential levels and alternating temperature regimes (Table 1) for each population and were fitted using a three-parameter sigmoidal model (Eq 2) to understand different water stress adaptability of the GR and GS populations in different thermal conditions (Fig 1).

All the populations exhibited a 100% reduction in germination at the lowest osmotic potential level (-1.6 MPa) at all alternating temperature regimes (Table 1; Fig 1). At the two sub-optimum thermal conditions (15/5°C and 35/25°C), both GR (SGW2 and CP2) and GS (Ch and SGM2) populations displayed a 100% reduction in germination at the second-lowest osmotic potential level (-0.8 MPa). At the same time, the two GS populations showed 2% and 3% (for Ch and SGM2, respectively) germination at the optimum alternating temperature regime (25/15°C) at -0.8 MPa osmotic potential, demonstrating the ability of the two GS populations to germinate even in high water stress conditions than the GR populations. However, the ability to germinate at higher water stress conditions does not imply higher water stress adaptability of the plants. Water stress adaptability can rather be understood by observing a 50% reduction in germination corresponding to osmotic potential treatment (*x0)* (Table 2).

**Table 2:** Estimated parameters of a three-parameter sigmoidal model $\left[(f=a/(1+exp{\left(\frac{x}{x0}\right)}^{b})\right]$ fitted to the germination data of two glyphosate-susceptible (Ch and SGM2) and two glyphosate-resistant (SGW2 and CP2) populations of *Chloris virgata* in response to different alternate day/light temperature regimes and osmotic potential levels.

Temperature (°C)	Populations	*a*	*b*	*x0* (MPa)	*R*^*2*^
15/5	Ch	101 ± 6	-0.09 ± 0.02	-0.24 ± 0.03	0.98
	SGM2	100 ± 5	-0.11 ± 0.02	-0.27 ± 0.03	0.99
	SGW2	100 ± 3	-0.09 ± 0.01	-0.29 ± 0.01	0.99
	CP2	100 ± 11	-0.16 ± 0.07	-0.23 ± 0.07	0.91
25/15	Ch	104 ± 10	-0.14 ± 0.05	-0.52 ± 0.08	0.97
	SGM2	102 ± 4	-0.14 ± 0.02	-0.45 ± 0.03	0.99
	SGW2	103 ± 8	-0.17 ± 0.05	-0.35 ± 0.06	0.97
	CP2	104 ± 7	-0.14 ± 0.04	-0.47 ± 0.05	0.98
35/25	Ch	101 ± 2	-0.12 ± 0.01	-0.39 ± 0.01	0.99
	SGM2	101 ± 5	-0.13 ± 0.03	-0.29 ± 0.03	0.98
	SGW2	103 ± 9	-0.15 ± 0.05	-0.43 ± 0.07	0.97
	CP2	103 ± 7	-0.16 ± 0.04	-0.39 ± 0.06	0.98

Abbreviations: ‘*a’* is an asymptote of the curve, ‘*b’* is the slope of the regression curve, ‘*x0’* is the osmotic potential level corresponding to 50% germination reduction and ‘*R*^*2*^*’* is a coefficient of determination. Values after ± are standard errors of the mean.

The ‘*x0’* values of all four populations were highest at the lowest alternating temperature regime (15/5°C), compared to the two higher temperature regimes (25/15°C and 35/25°C). The values of ‘*x0’* were ranging from -0.23 MPa to -0.29 MPa for all populations at 15/5°C compared to the lower *‘x0’* values at the higher alternating temperature regimes 25/15°C and 35/25°C (ranging from -0.35 MPa to -0.52 MPa at 25/15°C and from -0.29 MPa to -0.43 MPa at 35/25°C). These results suggest that at low temperatures, water stress adaptability of *C*. *virgata* declined and therefore the moisture requirement for germination of *C*. *virgata* is lower at a higher temperature. Therefore, at optimum thermal conditions, this weed species can germinate in areas where a limited quantity of soil moisture is available. Due to the ability of *C*. *virgata* to germinate in moisture stress conditions, this weed species could proliferate efficiently in the areas where summer fallow conditions have been in practice, even if light rainfall occurs. It is important to note that the seed production potential of *C*. *virgata* (>40,000 seeds plant^-1^) [1] could enhance the seed bank in the summer months.

The GR population SGW2 possessed the lowest *‘x0’* value (indicating higher water stress adaptability) at two sub-optimal temperature conditions (15/5°C and 35/25°C) as compared to other populations (Table 2). On the other hand, the SGW2 population exhibited the highest *‘x0’* value (indicating lower water stress adaptability) at the optimum temperature regime (25/15°C) compared to other populations. Moreover, the other three populations also displayed varying water stress adaptability under different alternating thermal conditions. Population CP2 showed similar characteristics to the GS populations (Ch and SGM2) in terms of water stress adaptability despite having the GR status. Therefore, no distinctive behavior was observed in terms of the water stress adaptability of the GS and GR populations. All the populations might have faced different agronomic practices in their initial generations for a longer period and that could be the reason for varying water stress adaptability under different alternating temperature regimes [14]. In the current study, all populations were grown in the same environment at Gatton, Queensland. However, there is limited knowledge on the effect of maternal environments and agronomic practices (followed at the seed collection sites) on *C*. *virgata* germination ecology.

*Chloris virgata* is characterized as a summer annual grass species [1]. Previous research on the germination response of a Queensland population of *C*. *virgata* to alternate temperature regimes suggested 30/20°C as the ideal temperature for optimum germination [20]. The same study also suggested that germination was lowest at a 15/5°C temperature regime. In the current study, all populations exhibited considerable germination percentages, even at the lowest alternating temperature regime (15/5°C) and 0 MPa osmotic potential (61%, 87%, 49%, and 47 for Ch, SGM2, SGW2, and CP2, respectively) (Table 1). These results suggest that *C*. *virgata* has the ability to germinate in winter months, despite being a summer annual. Therefore, Queensland’s farmers have been observing *C*. *virgata* throughout the year (Chauhan, personal observations). This could be because of a “weed shift” (transformation in compositions by weed populations due to agronomic practices) [25]. This type of the ‘weed shift’ behavior has previously been observed in many weed species such as *Sonchus oleraceus* L. [26], *Sisymbrium thellungii* O. E. Schulz. [27] and *Conyza bonariensis* L. [28]. However, it should not be implied that successful germination at low temperatures in laboratory conditions means successful survival in field conditions, as frost may kill weed species in winter. Therefore, future research experiments should focus on *C*. *virgata* phenology, in which the weed is planted at intervals to explore year-round survivability.

**Fig 2:**
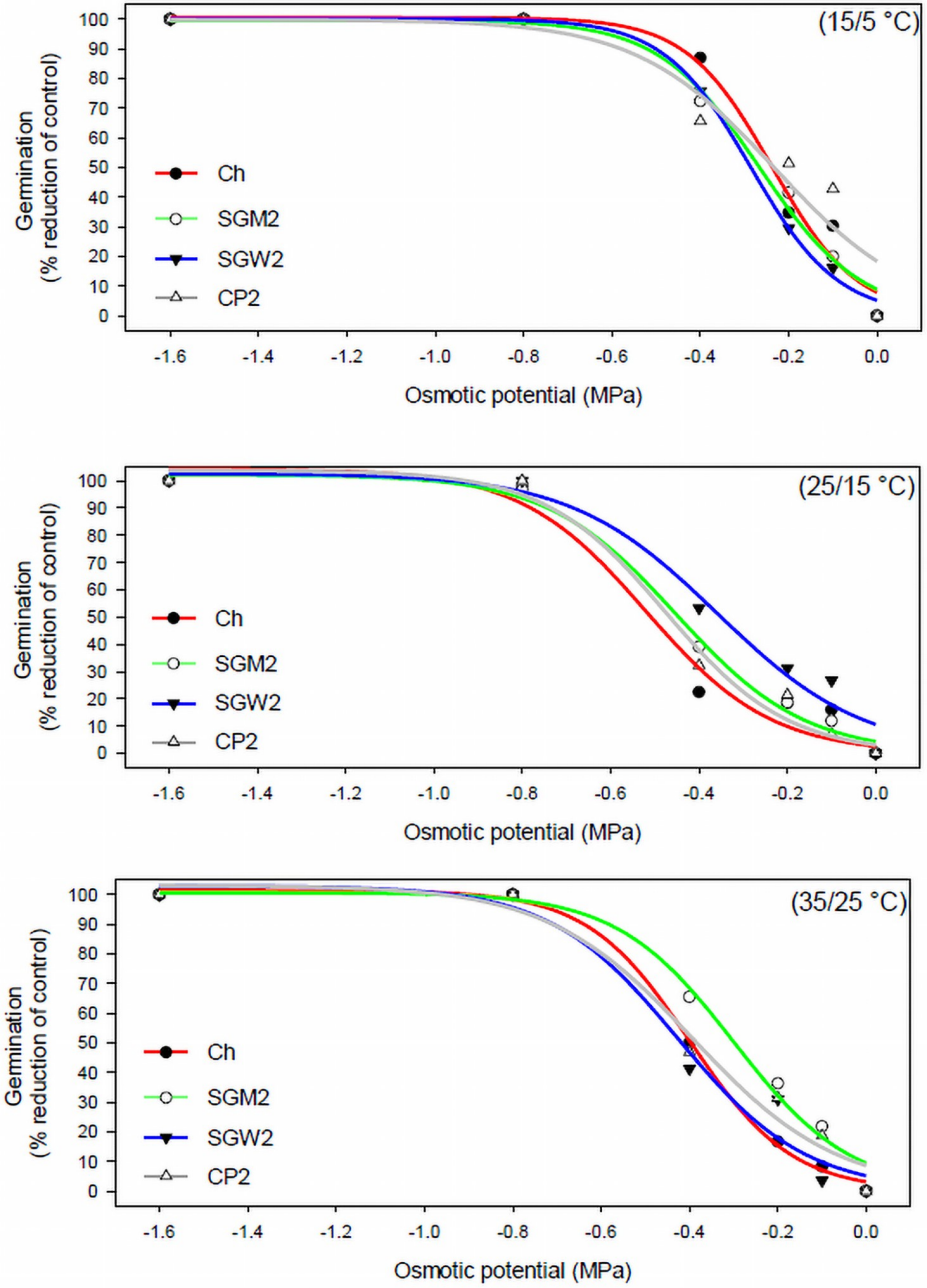
Monthly weather conditions of three locations from where the four populations of *Chloris virgata* were collected. (A) Monthly mean maximum and minimum temperature and (B) monthly mean rainfall. Ch was collected from Chinchilla; CP2 from Cecil Plains; and SGM2 and SGW2 from St George.

Seasonal weather parameters [mean maximum and minimum temperature (Fig 2A), and mean monthly rainfall (Fig 2B)] were acquired for the past 30 years from the Australian Bureau of Meteorology website for three locations from where the populations were collected (Fig 2) to understand monthly weather conditions. The mean minimum and maximum temperatures in summer months (November to February) and winter months (May to August) of all three locations resemble the alternating temperature regimes (15/5°C, 25/15°C, and 35/25°C) used in this study (Fig 2A). All three locations (Cecil Plains, Chinchilla, and St. George) exhibited summer dominant rainfall (Fig 2B); however, some amount of rainfall was recorded in the winter months as well.

All populations used in this study were collected from different agroecosystems [4] and possessed more than 40% germination at 0 MPa osmotic potential at the lowest alternating temperature regime (15/5°C). Therefore, as discussed earlier, all populations can germinate in the winter months if rainfall occurs. This could hinder different agricultural industries of the regions from where all the populations were gathered, for instance, the beef and pork industries in Chinchilla and Saint George regions by degrading pasture land, grain industries of the Saint George region by competing with winter grain crops. Also, the Cecil Plains region is considered one of the most fertile regions in terms of cotton production in the Southern hemisphere and the CP2 population exhibited more than 40% germination at two higher alternating temperature regimes (25/15°C and 35/25°C), demonstrating the ability to germinate in summer months and compete with cotton crops of the Cecil Plains region.

Three more biological parameters (T-parameters) (T10, T50, and T90) were acquired from R software using ‘SeedCalc’ package to further understand germination patterns of all the populations at three alternating temperature regimes and different osmotic potential levels, whereas T10, T50, and T90 represent incubation period (hours) required to attain 10% germination, 50% germination, and 90% germination, respectively (Table 3). The two lowest osmotic potential levels (-0.8 and -1.6 MPa) were excluded for T-parameters because negligible or no germination was observed at all alternating temperature regimes (Table 1).

**Table 3:** Biological parameters (T10, T50, and T90) of the four populations of *Chloris virgata*.

Temperature (°C)	Osmotic Potential (MPa)	T10 (h)				T50 (h)				T90 (h)			
		Ch	SGM1	SGW2	CP2	Ch	SGM1	SGW2	CP2	Ch	SGM1	SGW2	CP2
15/5	0	98.9 ± 0.3	107.0 ± 2.4	138.4 ± 2.2	128.0 ± 2.1	110.6 ± 1.4	197.1 ± 1.7	204.2 ± 0.9	155.3 ± 5.7	152.4 ± 18.4	315.4 ± 34.3	341.4 ± 45.8	188.0 ± 12.4
15/5	-0.1	100.6 ± 0.7	128.8 ± 5.8	190.0 ± 15.1	129.2 ± 2.8	136.0 ± 12.0	222.3 ± 20.1	244.0 ± 5.3	237.3 ± 27.8	252.0 ± 26.0	325.7 ± 60.8	499.2 ± 44.4	348.0 ± 48.0
15/5	-0.2	120.4 ± 6.1	129.5 ± 3.1	256.8 ± 9.6	201.6 ± 35.8	188.0 ± 8.7	240.0 ± 18.3	377.6 ± 27.1	384.0 ± 63.0	303.6 ± 8.9	513.0 ± 20.8	615.2 ± 20.2	583.6 ± 14.8
15/5	-0.4	365.8 ± 44.4	142.4 ± 40.0	445.6 ± 29.2	385.6 ± 34.4	436.0 ± 41.9	252.0 ± 46.6	566.0 ± 30.8	510.7 ± 30.8	532.3 ± 61.7	606.6 ± 20.0	630.0 ± 0.7	630.4 ± 31.3
25/15	0	50.6 ± 0.01	50.6 ± 0.1	50.7 ± 0.2	50.7 ± 0.1	60.9 ± 0.04	60.7 ± 0.4	61.6 ± 1.2	61.3 ± 0.8	71.2 ± 0.1	70.9 ± 0.7	84.6 ± 14.1	78.3 ± 7.7
25/15	-0.1	51.0 ± 0.3	51.7 ± 0.2	55.7 ± 1.8	52.5 ± 0.3	62.9 ± 1.5	66.4 ± 1.2	88.1 ± 6.2	77.1 ± 5.1	88.7 ± 10.1	107.5 ± 7.9	132.7 ± 4.6	146.8 ± 19.6
25/15	-0.2	51.5 ± 0.7	52.6 ± 0.1	59.2 ± 2.9	53.7 ± 0.9	70.1 ± 8.1	75.9 ± 5.3	95.9 ± 6.3	94.7 ± 13.3	104.7 ± 23.0	146.0 ± 9.9	152.8 ± 7.1	270.4 ± 11.0
25/15	-0.4	58.8 ± 2.5	78.7 ± 0.3	96.8 ± 11.8	69.9 ± 10.8	122.0 ± 19.1	112.0 ± 2.0	181.0 ± 21.5	177.0 ± 11.4	379.2 ± 22.6	240.4 ± 20.6	443.8 ± 14.3	323.2 ± 15.8
35/25	0	50.4 ± 0.0	50.4 ± 0.0	50.5 ± 0.1	50.5 ± 0.1	60.5 ± 0.9	60.0 ± 0.0	60.0 ± 0.0	60.4 ± 0.7	69.6 ± 0.0	69.6 ± 0.0	71.2 ± 1.6	70.3 ± 0.7
35/25	-0.1	51.3 ± 0.2	50.7 ± 0.2	50.6 ± 0.1	50.6 ± 0.1	60.9 ± 0.8	61.5 ± 1.4	64.6 ± 1.8	61.0 ± 0.9	113.6 ± 24.9	82.4 ± 7.0	71.9 ± 1.4	79.2 ± 6.3
35/25	-0.2	54.7 ± 2.7	51.1 ± 0.1	51.2 ± 0.6	51.4 ± 0.2	64.1 ± 4.8	63.8 ± 0.6	83.8 ± 19.5	64.7 ± 2.2	282.7 ± 13.2	120.4 ± 16.6	322.4 ± 12.9	108.8 ± 11.1
35/25	-0.4	51.4 ± 0.1	67.5 ± 9.8	98.1 ± 41.0	55.2 ± 2.4	64.9 ± 1.0	115.5 ± 22.4	178.0 ± 49.0	105.7 ± 47.5	237.1 ± 19.0	268.0 ± 74.9	482.4 ± 54.5	449.6 ± 14.7

Abbreviation: Incubation period required to attain 10%, 50%, and 90% germination for T10, T50, and T90, respectively; (h) represents the hour and; values after ± are standard errors of the mean. Glyphosate-susceptible populations (Ch and SGM2) and glyphosate-resistant populations (SGW2 and CP2).

Time of germination initiation (T10) for all populations at all the alternating temperature regimes increased alongside increasing moisture stress levels (Table 3) and this trend remained similar for the incubation period required to attain 50% and 90% germination (T50 and T90, respectively) of all populations (Table 3). These outcomes are clear indications of delayed water imbibition by the seeds of *C*. *virgata* in increasing water stress conditions.

In addition, the GR population SGW2 exhibited a distinct pattern for T10, T50, and T90 at all alternating temperature regimes and osmotic potential levels (Table 3). To elaborate, the GR population SGW2 required a longer incubation time to reach 10%, 50%, and 90% germination at all alternating temperature regimes and osmotic potential levels as compared to other populations. The SGW2 population required 138 hours to initiate the germination process (T10) at 15/5°C (osmotic potential 0 MPa), which was higher than other populations at the same temperature and osmotic potential, and it was similar for T-parameters (T10, T50, and T90) at all alternating thermal conditions and osmotic potential levels.

The reduced germination percentage and delayed or extended germination characteristics of the GR population SGW2, especially at the highest alternating temperature regime (35/25°C), might be the expression of an escape mechanism against pre-planting weed management operations (e.g., applications of burndown herbicides) in summer fallow conditions and this particular population could guard itself against pre-plating burndown herbicide application; therefore, securing successful survival. The SGW2 population also exhibited an ability to germinate at the lowest alternating temperature regime (15/5°C) and thus may be capable of germinating in winter months (Table 1) and competing with winter grains grown in the Saint George region, such as wheat and barley.

Consequently, the SGW2 population could become considerably hard to control in winter cereals. Pinoxaden, an ACCase inhibitor herbicide, is recommended to control grass weed species in winter cereals and it is the only chemical formulation available in the market to target grass weeds in winter cereals. However, our previous research suggested that pinoxaden was not effective on all the four populations used in this study [4]. This might cause difficulties in selecting herbicides in order to control *C*. *virgata* with other multiple weed species in the same fields in winter cereals.

Results from the current study have direct implications for the development of integrated weed management programs, selection of tillage timing, choosing a proper timing for herbicide application to optimize efficacy, and adjusting crop calendars to achieve ecological based control of this species. Data from this study could also aid in the development of resistance simulation models to forecast the advancement and population dynamics of GR *C*. *virgata*.

## Conclusion

The adaptability to varying temperature regimes and osmotic potential levels of the two GR (SGW2 and CP2) and two GS (Ch and SGM2) populations of *C*. *virgata* revealed in this study indicates the potential of this species to infest cropping regions and fallows of the south-east region of Queensland, Australia. This study also stipulated the need for developing site-specific weed control tactics rather than species-specific control measures, especially for GR populations of *C*. *virgata*. We acknowlede that the results of this study cannot be generalized because only a limited number of GR and GS populations were used. Moreover, very often GR populations coexist with GS populations, and therefore replacement series experiments may provide detailed information on their relative competiveness. An ability of *C*. *virgata* to exhibit considerable germination at the lowest alternating temperature regimes (15/5°C) demonstrates its year-round emergence possibilities, despite the summer annual life cycle, thus enriching seed banks and ensuring infestation in subsequent years. This study suggests several research gaps that need to be addressed in the future to achieve efficient control of *C*. *virgata* such as weed phenology, glyphosate-alternatives, the efficacy of herbicides in moisture stress conditions, and optimizing the benefits from tillage operations.
